# 중환자실 간호사의 표준주의 수행에 영향을 미치는 요인: 서술적 상관관계 연구

**DOI:** 10.7586/jkbns.26.016

**Published:** 2026-05-26

**Authors:** Hana Seo, Nahyun Kim

**Affiliations:** 1 Surgical ICU, Pusan National University Hospital, Busan, Korea; 1 부산대학교병원 외과계 중환자실; 2 Department of Nursing, Graduate School of Keimyung University, Daegu, Korea; 2 계명대학교 대학원 간호학과; 3 College of Nursing, Keimyung University, Daegu, Korea; 3 계명대학교 간호대학

**Keywords:** Infection control, Intensive care unit, Nurses, Organizational culture, Universal precautions, 감염관리, 중환자실, 간호사, 조직문화, 표준주의

## Abstract

**Purpose:**

This descriptive correlational study aimed to identify personal, organizational, and environmental factors influencing the performance of standard precautions (SPs) among intensive care unit (ICU) nurses.

**Methods:**

A structured questionnaire survey was conducted among 166 ICU nurses at two tertiary hospitals in South Korea. Data were analyzed using IBM SPSS Statistics version 25.0, including independent t-tests, analysis of variance, Pearson correlation analysis, and hierarchical regression analysis.

**Results:**

Hierarchical regression analysis identified organizational culture for infection control (β = .41, *p* < .001), awareness of SPs (β = .37, *p* < .001), and clinical experience (β = .18, *p* = .007) as significant predictors of SP performance. In contrast, the infection prevention environment was not a statistically significant predictor. The final model explained 54.0% of the variance in SP performance (F = 23.39, *p* < .001), with an adjusted R^2^ of 52.0%.

**Conclusion:**

To enhance SP performance among ICU nurses, multidimensional, career-tailored interventions are needed to move beyond traditional knowledge-based education and facilitate the transition from awareness to practice. In addition, establishing supportive and constructive communication systems within a nonpunitive environment for reporting infection control errors or noncompliance, together with supportive leadership, may help create a sustainable organizational foundation for infection prevention. Ultimately, shifting the focus from physical facilities to organizational culture is essential for preventing pathogen transmission and ensuring physiological safety.

## 서론

### 1. 연구의 필요성

의료환경에서 발생하는 의료관련감염의 상당 부분은 의료진의 손과 환경에 기인한 것으로 알려져 있으며[1] 특히 중환자실은 일반병동에 비해 의료관련감염 발생률이 약 2.7배 높은 것으로 보고된다[2]. 이는 의료비용 증가와 재원기간의 연장, 그리고 환자의 사망률을 증가시키는 중대한 임상적 문제로 철저한 관리가 요구된다[1,2]. 특히 중환자실 환자는 면역 기능이 저하된 상태에서 각종 침습적 처치가 반복적으로 이루어져 의료관련감염에 대한 생물학적 취약성이 높으며 감염 발생 시 중증 합병증으로 이어질 가능성이 크다[2]. 따라서 고위험 병원체의 생물학적 전파를 예방하기 위한 활동은 중환자실 간호에서 높은 우선순위로 다루어져야 한다.

표준주의는 의료기관 내에서 의료관련감염을 예방하기 위한 기본 방침으로 환자의 혈액, 체액을 비롯한 모든 분비물과 배설물을 잠재적 감염원으로 간주하는 개념이다[3]. 표준주의에는 손위생, 환경관리, 개인보호구, 안전한 주사행위, 호흡기 예절 등 다양한 감염예방 활동이 포함되며[3,4] 이를 준수하는 것은 의료진과 환자를 보호하기 위한 필수적인 실무이다[2]. 그럼에도 불구하고 표준주의 이행 수준은 여전히 충분하지 못하다는 주장이 제기되고 있어[5] 의료관련감염 예방을 위한 실질적인 개선 노력이 필요함을 시사하고 있다.

선행연구에 따르면 국내 간호사의 표준주의 준수율은 약 60%∼97%로 보고되며[6,7] 의료기관의 형태나 측정 도구에 따라 차이를 보이는 것으로 나타났다[6,8]. 간호사의 표준주의 수행은 다양한 요인의 영향을 받으며, 이러한 요인은 크게 개인적 요인, 조직적 요인, 환경적 요인으로 구분할 수 있다[7-13]. 이 중 개인적 요인은 행동 주체의 내적 특성으로서 개인이 특정 행동을 수행할지를 결정하는 일차적인 인지적 근거가 된다[9]. 표준주의 수행과 관련된 개인적 요인으로는 임상근무경력과 같은[6,14-16] 인구사회학적 특성과 인지도[17,18], 지식[14,15,18,19] 등의 인지적 특성이 주요하게 보고되고 있다. 선행연구에 따르면 이러한 인지적 특성은 표준주의 수행도를 높이는 주요한 변수이나[6,14,15,18,19], 실제 간호사의 인지적 수준이 높음에도 수행도는 이에 미치지 못한다는 결과가 지속적으로 보고되고 있다[5,6,18,19]. 이는 간호사가 보유한 인지적 특성이 실제 행동으로 충분히 전환되지 못하고 있음을 의미한다. 이에 개인적 요인 중 하나인 인지도를 분석에 포함하여 인지와 행동의 불일치가 나타나는 임상 현장의 실태를[17,18,20] 분석해보고자 한다.

조직적 요인은 의료기관 내에서 의료진의 행동을 조절하고 지원하는 상위의 구조적 체계를 의미한다[21]. 그 중 조직문화는 조직 내부의 가치관, 규범, 신념 등의 총체적 특성으로[22] 조직구성원의 정체성을 형성하고 실제 행동에 영향을 미치는 결정적 요인으로 작용하는 것으로 보고된다[6,22,23]. 감염관리 조직문화는 이러한 조직문화의 보편적인 개념을 환자안전과 의료관련감염 예방 차원에서 구체화하고 보완한 개념으로[12] 관리자의 태도, 조직의 지원 그리고 조직 내 의사소통 등을 포함한다[12,24]. 이러한 조직적 요인은 간호사의 표준주의 수행에 유의한 영향을 미치는 것으로 보고되며[6,13,23-25], 특히 조직 내 의사소통이 활발하거나 조직구성원이 감염관리를 중요시하는 조직문화일수록 표준주의 수행이 강화되는 것으로 나타났다[24]. 이는 감염관리 조직문화가 간호사의 표준주의 수행을 촉진하거나 저해할 수 있는 중요한 요인임을 시사하며 coronavirus disease 2019 (COVID-19) 이후 변화된 의료환경에서 감염관리 조직문화가 간호사의 표준주의 수행에 미치는 영향을 재확인할 필요가 있다.

또한 환경적 요인은 의료환경에서 실무 수행에 필요한 물적 자원의 구비, 시설의 가용성 등을 포함하는 개념이다[21]. 그 중 감염예방환경은 의료기관 내 적절한 설비와 장비의 제공[26], 업무와 인력관리[27], 감염관리지침, 개인보호구, 소독 장비 등 조직적 지원과 물리적 지원을 포괄하는 개념으로[10] 간호사의 표준주의 수행과 관련이 있는 것으로 보고되어 있다[10,11,13,15,28,29]. COVID-19 이후 개인보호구 사용의 확대와 감염관리지침 강화 등 의료환경 전반에 변화가 나타나면서 감염예방환경의 중요성은 더욱 강조되고 있다[26]. 그러나 국외 연구에서는 여전히 장비의 부족 문제가 보고되고 있으며[30], 강화된 제도적, 물리적 환경으로 인한 업무 부담 증가가 오히려 표준주의 수행을 저해할 수 있다는 결과도 제시되고 있다[20]. 그럼에도 불구하고 물리적·제도적 환경의 구축은 인지 수준을 실제 수행으로 이행하기 위한 필수 전제 조건이다[28]. 따라서 환경적 요인이 개인적, 조직적 요인과 어떻게 상호작용하며 간호사의 표준주의 수행에 영향을 미치는지를 통합적으로 분석할 필요가 있다.

의료관련감염은 예방이 가능한 영역이므로, 중재 가능한 요인을 규명하고 이를 개선하기 위한 지속적인 노력이 필수적이다[2]. 특히 많은 의료관련감염은 표준주의를 준수함으로써 예방할 수 있는 것으로 보고되었으며[4] 최근 의료환경에서 그 중요성이 더욱 강조되고 있다[26]. 간호사는 의료기관 내 의료종사자 중 가장 많은 직접 의료행위를 제공하는 직군으로[31] 고위험 병원체에 노출되거나 전파시킬 위험이 높은 집단이다[5]. 이에 따라 간호사를 대상으로 감염관리 수행과 관련된 다양한 연구들이 수행되어 왔다[5-8,10-20,23-25,27-31]. COVID-19 이후 간호사의 표준주의 수행 영향 요인에 대한 국내 연구는 늘어난 추세이나[8,32] 대다수의 연구가 개인적 요인이나 조직적 요인 등 제한된 측면에서 분석되었다는 한계점이 보고된다[32]. 반면 국외 연구에서는 간호사의 표준주의 수행을 저해하는 요인으로 시간 부족, 업무량 과다, 개인의 판단과 같은 다양한 요인을[5,20] 보고하고 있다. 이러한 맥락에서 국내 의료환경의 특수성과 변화된 임상 현장을 반영하여 간호사의 표준주의 수행에 영향을 미치는 다각적 요인간의 관계를 분석하는 연구가 요구되고 있다[8]. 이에 본 연구는 대상자의 일반적 특성을 기저에 두고 행동의 일차적 근거가 되는 개인적 요인을[9] 우선적으로 파악하고자 한다. 이어 조직구성원의 정체성과 행동에 영향을 미치는 조직적 요인과[12] 실무 수행의 구조적 토대인 환경적 요인을[21] 순차적으로 포함하여 고찰의 범위를 확장한다. 최종적으로 개인적, 조직적, 환경적 측면의 다각적인 접근을 통해 중환자실 간호사의 표준주의 수행에 영향을 미치는 요인을 통합적으로 분석하고자 한다.

### 2. 연구의 목적

본 연구의 목적은 상급종합병원 중환자실 간호사를 대상으로 표준주의 수행에 영향을 미치는 다각적인 요인을 분석하고 주요 영향 요인을 규명하는 것이다. 구체적인 목적은 다음과 같다.

1) 대상자의 표준주의 인지도, 감염관리 조직문화, 감염예방환경 및 표준주의 수행도를 파악한다.

2) 대상자의 일반적 특성에 따른 표준주의 인지도, 감염관리 조직문화, 감염예방환경 및 표준주의 수행도 차이를 분석한다.

3) 대상자의 표준주의 수행도에 영향을 미치는 요인을 확인한다.

## 연구 방법

### 1. 연구 설계

중환자실 간호사를 대상으로 표준주의 수행에 영향을 미치는 요인을 파악하기 위해 시도된 서술적 상관관계 연구이다. 본 연구는 STROBE (Strengthening the Reporting of Observational Studies in Epidemiology) Statement 지침을 참고하여 보고하였다.

### 2. 연구 대상

연구대상은 부산광역시와 대구광역시의 상급종합병원 두 곳에 근무하는 중환자실 간호사이며 편의표집 방식으로 대상자를 모집하였다. 연구대상자 선정 시 임상 경력에 대한 제한은 두지 않았으며 직접 간호를 수행하지 않고 단위 부서의 관리와 행정 업무를 전담하는 수간호사 이상의 관리자는 연구 대상에서 제외하였다. 연구대상자의 수는 선행연구[10,28]를 참고하여 G*Power (version 3.1.9.7, Heinrich-Heine-Universität Düsseldorf, Düsseldorf, Germany)를 이용하여 산출하였다. 위계적 회귀분석을 위해 유의수준(α)= .05, 검정력(1-β)= .90, 중간 효과 크기(f^2^)= .15, 예측 인자 12개를 고려하여 최소 155명이 필요한 것으로 산출되었다. 탈락율 약 10%를 적용하여 171부의 설문지를 배부하였고, 이 중 응답이 불성실한 5부를 제외한 총 166명의 자료를 최종 분석에 사용하였다.

### 3. 연구 도구

연구대상자의 일반적 특성은 선행연구를[10,28] 참고하여 9개 문항으로 구성하였다. 구체적인 내용은 성별, 연령, 결혼 여부, 최종 학력, 임상근무경력, 현 근무부서, 일평균 담당 환자 수, 일평균 근무시간과 감염원에 노출된 경험의 유무이다.

#### 1) 표준주의 인지도와 수행도

표준주의 인지도와 수행도는 Hong과 Park [33]이 간호대학생을 대상으로 개발 후 Baek [28]이 간호사의 표준주의 수행 측정을 위해 수정한 도구의 사용 승인을 받은 뒤 활용하였다. 일부 문항의 내용은 본 연구자가 ‘의료관련감염 표준예방지침’을[4] 반영하여 수정하였다. 주요 수정 내용은 첫째, 환경관리 영역의 실무 변화를 반영하였다. 과거 간호사가 직접 수행하던 의료환경 청소와 소독 업무가 현재는 환경관리 직원을 감독 및 관리하는 형태로 전환된 점을 고려하여 문항을 수정하였다. 둘째, 직원안전 영역에서 심폐소생술 상황에서의 개인보호구 착용 지침을 구체화하였다. 셋째, 설문 문항의 용어를 ‘의료관련감염 표준예방지침’과[4] 통일하여 일관성을 높였다. 수정된 도구는 간호학 교수 1인, 상급종합병원 감염관리실무전문가 1인, 상급종합병원 감염관리실 팀장 1인, 중환자 전문간호사 1인, 간호학 박사 1인 등 총 5인의 전문가로부터 내용타당도(Content Validation Index) 검증을 거쳤다. 모든 문항의 내용타당도 값이 .80 이상으로 수정된 도구가 표준주의 인지도와 수행도를 측정하기에 타당한 수준임을 확인하였다. 이후 문항 이해도와 응답 용이성을 확인하기 위해 중환자실 간호사 5인에게 예비 조사(pilot study)를 실시하여 모든 문항이 임상 현장에서 혼동 없이 이해됨을 확인하였다.

본 도구는 39개의 문항으로 구성되었으며 동일한 문항에 대해 표준주의 인지도와 수행도를 각각 응답하도록 함으로써 두 영역을 측정하였다. 하위 영역은 총 9개로 손위생 10문항, 개인보호구 착용 9문항, 환자 물품 관리와 주변 장비 2문항, 세탁물 관리 2문항, 직원안전 4문항, 환자의 적절한 배치 2문항, 환경관리 2문항, 호흡기 예절 3문항, 안전한 주사행위 5문항으로 구성된다. 표준주의 인지도는 대상자가 각 항목의 중요성을 자각하고 있는 정도를 확인하기 위해 ‘전혀 중요하지 않다’ 1점에서 ‘매우 중요하다’ 5점의 Likert 5점 척도를 활용하였다. 표준주의 수행도는 대상자의 표준주의 수행 정도를 확인하기 위해 ‘전혀 수행하지 않는다’ 1점부터 ‘항상 수행한다’ 5점의 Likert 5점 척도로 구성하였다. 표준주의 인지도와 수행도의 점수가 높을수록 표준주의에 대한 인지와 수행 정도가 높음을 의미한다. 본 연구에서 사용한 표준주의 인지도와 수행도의 구체적인 문항은 Appendix 1과 Appendix 2에서 제시하였다. 본 도구는 Baek [28]의 연구에서 표준주의 수행도 측정 당시 Cronbach’s ⍺는 .89이었으며 본 연구에서 표준주의 인지도 측정에서 Cronbach’s ⍺는 .95, 표준주의 수행도 측정에서 Cronbach’s ⍺는 .94이었다.

#### 2) 감염관리 조직문화

감염관리 조직문화는 미국 보건의료연구소(Agency for Healthcare Research and Quality)의 조직문화 측정도구를[34] 기반으로 Park [35]이 환자안전문화 측정도구로 수정 후 Moon과 Jang [12]이 감염관리지침 수행에 대한 조직문화 측정을 위해 수정한 도구의 사용 승인을 받은 후 활용하였다. 감염관리 조직문화는 총 10문항으로 구성된 도구로 Moon과 Jang [12]의 연구에서 Likert 7점 척도를 사용하였다. 본 연구에서는 응답자의 인지적 부담과 점수 분포의 편향을 최소화하기 위해[36] Likert 5점 척도로 수정하여 측정하였으며, 각 문항은 ‘전혀 그렇지 않다’ 1점에서 ‘매우 그렇다’ 5점으로 구성된다. 점수가 높을수록 감염관리 조직문화를 긍정적으로 인식함을 의미한다. 수정된 도구는 개념적 기틀을 유지하며 척도만을 변경한 후 중환자실 간호사 5인을 대상으로 예비 조사를 실시하여 문항 이해도와 응답 용이성을 검토하였다. 조사 결과 모든 문항의 가독성이 적절하고 임상 현장 적용에 혼동이 없음을 확인하였다. Moon과 Jang [12]의 연구에서 Cronbach’s ⍺는 .85이며 본 연구에서 Cronbach’s ⍺는 .85이었다.

#### 3) 감염예방환경

감염예방환경은 Han과 Moon [37]이 방사선사를 대상으로 개발하고 Ahn 등[11]이 응급실 간호사에게 사용할 목적으로 수정한 도구에 대해 사용 승인을 받은 후 사용하였다. 본 연구에서는 최신 임상 현장을 반영하여 일부 문항을 수정하였다. 구체적으로, 개별 부서에서 수행되던 소독 및 멸균 업무가 중앙공급실로 전문화되며 이관된 실무 현황을 반영하여 ‘소독과 멸균’에 대한 문항을 간호사가 직접 수행하는 ‘환경청소 및 소독을 위한 도구와 물품’에 대한 문항으로 변경하였다. 수정된 도구는 간호학 교수 1인, 상급종합병원 감염관리실무전문가 1인, 상급종합병원 감염관리실 팀장 1인, 중환자 전문간호사 1인, 간호학 박사 1인 등 총 5인의 전문가로부터 내용타당도 검증을 받았다. 모든 문항의 내용타당도 값이 .80 이상으로 감염예방환경 인식 정도를 측정하는데 타당한 도구임을 확인하였다. 이후 중환자실 간호사 5인에게 예비 조사를 실시하여 실제 중환자실 간호사가 응답하기에 무리가 없음을 파악하였다.

감염예방환경 측정 도구는 총 11문항으로 각 문항은 ‘전혀 그렇지 않다’ 1점에서 ‘매우 그렇다’ Likert 5점의 척도로 구성되었으며 점수가 높을수록 감염예방환경이 잘 조성되어 있음을 의미한다. Ahn 등[11]의 연구에서 Cronbach’s ⍺는 .85이었으며 본 연구에서 Cronbach’s ⍺는 .86이었다.

### 4. 자료 수집

자료수집 전 연구 대상인 부산광역시와 대구광역시 상급종합병원의 간호부에 연구의 목적과 내용, 절차를 설명하고 자료수집에 대한 공식적인 허락을 받은 후 연구를 진행하였다. 연구책임자는 각 병원의 중환자실을 직접 방문하여 지정된 장소에 ‘연구대상자 모집 공고문’을 게시하였다. 2023년 12월 5일부터 12월 30일까지의 설문조사 기간 동안 연구책임자가 각 중환자실 간호사실을 방문하여 자발적으로 연구 참여 의사를 밝힌 대상자에게 설문지를 배부하였다. 이후 응답이 완료된 설문지는 연구대상자가 직접 개별 불투명 봉투에 넣어 밀봉한 뒤 간호사실 내 지정된 수거함에 투입하도록 하였으며 이를 연구책임자가 수거하였다.

### 5. 자료 분석

수집한 자료는 통계프로그램 SPSS version 25.0 (IBM Corp., Armonk, NY, USA)을 이용하여 분석하였다. 구체적인 분석 절차는 다음 과정과 같다.

1) 대상자의 일반적 특성을 실수와 백분율 그리고 평균과 표준편차를 산출하여 분석하였다.

2) 대상자의 표준주의 인지도, 감염관리 조직문화, 감염예방환경, 표준주의 수행도는 기술통계를 이용하여 평균과 표준편차를 확인하였다.

3) 대상자의 일반적 특성에 따른 표준주의 인지도, 감염관리 조직문화, 감염예방환경, 표준주의 수행도는 independent t-test와 ANOVA로 사후 검증은 Scheffé test를 시행하였다.

4) 표준주의 인지도, 감염관리 조직문화, 감염예방환경과 표준주의 수행도 간의 상관성은 Pearson correlation coefficient을 산출하여 분석하였다.

5) 표준주의 수행도에 영향을 미치는 요인을 분석하기 위해 위계적 회귀분석을 실시하였다.

### 6. 윤리적 고려

본 연구는 연구 윤리 원칙을 준수하였으며 연구대상자 보호를 위해 계명대학교 생명윤리위원회의 승인(40525-202308-HR-038-02)을 받은 후 진행하였다. 설문조사 시행 전 연구책임자는 연구대상자에게 연구 목적, 수집 자료의 비밀 보장, 관리 방법 및 활용범위를 포함한 연구설명문을 통해 충분한 설명을 제공하였다. 연구대상자가 자발적으로 참여를 결정한 후 서면으로 연구동의서를 작성하였으며, 원하는 경우 언제든지 연구 참여를 철회할 수 있고 참여 여부에 따른 어떠한 불이익도 없음을 안내하였다. 자료수집 시에는 연구대상자의 익명성을 보장하기 위해 개인 식별 정보를 수집하지 않았으며 자료수집 전 과정에서 응답 내용이 타인에게 노출되지 않도록 보안을 철저히 유지하였다. 수집된 모든 자료는 본 연구 목적 외에는 사용되지 않으며 생명윤리법 시행규칙 제 15조에 근거하여 연구 종료 시점으로부터 3년간 잠금장치가 있는 연구책임자의 개인 서랍에 보관 후 파쇄기를 이용하여 완전 폐기할 예정임을 사전에 고지하였다. 설문 응답을 완료한 대상자에게는 감사의 의미로 소정의 답례품을 제공하였다.

## 연구 결과

### 1. 연구대상자의 일반적 특성

연구대상자의 연령은 ‘30세 이상’이 86명(51.8%)으로 가장 많았으며 ‘30세 미만’은 80명(48.2%)이었다. 성별은 ‘여성’이 152명(91.6%)으로 대다수를 차지하였다. 결혼 여부는 ‘미혼’ 118명(71.1%), ‘기혼’ 48명(28.9%)으로 나타났다. 최종 학력은 ‘학사’ 학위 소지자가 135명(81.3%), ‘석사’ 21명(12.7%), ‘전문학사’ 10명(6.0%) 순이었다. 임상근무경력은 ‘60∼119개월’이 66명(39.8%)으로 가장 많았으며 ‘24∼59개월’ 51명(30.7%), ‘120개월 이상’ 30명(18.1%), ‘6∼23개월’ 19명(11.4%)이었다. 현 근무부서는 ‘외과계 중환자실’이 85명(51.2%)으로 가장 많았으며 ‘내과계 중환자실’ 61명(36.7%), ‘응급 중환자실’ 20명(12.1%) 순으로 확인되었다. 일평균 담당 환자 수는 ‘3명 미만’이 157명(94.6%)으로 대부분을 차지하였으며 ‘3∼4명’이 9명(5.4%)이었다. 일평균 근무 시간은 ‘8∼9시간’ 근무 집단이 151명(91.0%), ‘8시간 미만’ 근무 집단이 15명(9.0%)으로 나타났다. 감염원 노출 경험의 경우 ‘아니오’가 91명(54.8%)으로 ‘예’ 75명(45.2%)보다 다소 높게 확인되었다(Table 1).

### 2. 표준주의 인지도, 표준주의 수행도, 감염관리 조직문화와 감염예방환경의 정도

연구대상자의 표준주의 인지도는 5점 만점 기준 평균 4.80 ± 0.25점으로 나타났으며 최솟값은 3.92점이고 최댓값은 5점이었다(Table 2). 하위영역 별로는 안전한 주사행위가 4.93 ± 0.22점으로 가장 높았고 환자 물품 관리와 주변 장비 4.88 ± 0.28점, 직원안전 4.86 ± 0.29점, 환자배치 4.85 ± 0.33점 순으로 나타났다. 반면 환경관리 4.65 ± 0.49점, 세탁물관리 4.67 ± 0.48점은 상대적으로 낮은 점수를 보였다. 그 외 손위생은 평균 4.76 ± 0.30점, 개인보호구 4.81 ± 0.28점, 호흡기 예절 4.77 ± 0.40점으로 확인되었다. 표준주의 수행도는 5점 만점에 평균 4.56 ± 0.39점이었으며, 최솟값은 3.03점, 최댓값은 5점으로 나타났다. 하위영역 중 안전한 주사행위 4.88 ± 0.31점이 표준주의 인지도와 마찬가지로 가장 높았으며, 환자 물품 관리와 주변 장비 4.77 ± 0.44점, 직원안전 4.62 ± 0.47점, 환자배치 4.56 ± 0.63점, 개인보호구 4.54 ± 0.43점, 손위생은 평균 4.48 ± 0.51점, 세탁물관리 4.46 ± 0.66점, 환경관리 4.37 ± 0.81점, 호흡기 예절 4.29 ± 0.68점 순으로 확인되었다. 표준주의 인지도와 수행도의 구체적인 문항은 Appendix 1과 2에 제시하였다. 감염관리 조직문화는 5점 만점에 평균 4.02 ± 0.53점이었으며 최솟값은 2.80점, 최댓값은 5점이었다. 이 중 가장 점수가 낮은 문항은 ‘감염관리지침이 잘 지켜지지 않는다는 것을 알았을 때 부서에 자유롭게 의견을 제시한다(3.52점).’이었다(Appendix 3). 감염예방환경은 5점 만점에 평균 4.42±0.47점으로 최솟값은 3.27점, 최댓값은 5점이었다. 가장 점수가 낮은 문항은 ‘우리병원은 감염 예방교육을 정기적으로 실시한다(4.05점).’, 가장 높은 문항은 ‘감염노출 방어를 위한 개인보호구(마스크, 안면보호대, 글러브)가 다양하게 구비되어 있다(4.80점).’이었다(Appendix 4).

### 3. 일반적 특성에 따른 표준주의 수행도의 차이

표준주의 수행도는 결혼 여부, 최종 학력, 임상근무경력, 일평균 근무시간에서 유의한 차이가 확인되었다(Table 3). 결혼 여부에서 ‘기혼’(4.66 ± 0.33)이 ‘미혼’(4.51 ± 0.41)보다 표준주의 수행도가 유의하게 높음이 확인되었고(t = 2.26, *p* = .025), 최종 학력에서는 ‘석사’(4.75 ± 0.26)가 ‘학사’(4.55 ± 0.38), ‘전문학사’(4.30 ± 0.58)에 비해 높은 표준주의 수행도 점수를 보였다(F = 4.86, *p* = .009). 임상근무경력은 ‘120개월 이상’ 근무한 간호사(4.75 ± 0.24)가 ‘24∼59개월’(4.54 ± 0.46), ‘60∼119개월’(4.50 ± 0.35), ‘6∼23개월’(4.48 ± 0.42) 근무한 간호사보다 높은 점수가 나타났다(F = 3.30, *p* = .022). 일평균 근무시간은 ‘8시간 미만’ 근무 집단(4.83 ± 0.17)이 ‘8∼9시간’ 근무 집단(4.53 ± 0.40)보다 표준주의 수행도 점수가 유의하게 높았다(t = 5.54, *p* < .001).

### 4. 표준주의 인지도, 감염관리 조직문화, 감염예방환경과 표준주의 수행도 간 관계

표준주의 인지도, 감염관리 조직문화, 감염예방환경 및 표준주의 수행도 간 상관관계를 분석한 결과, 표준주의 수행도는 감염관리 조직문화(r = .58, *p* < .001), 표준주의 인지도(r = .55, *p* < .001), 감염예방환경(r = .52, *p* < .001)과 유의한 정적 상관관계를 보였다(Table 4).

### 5. 표준주의 수행도에 영향을 미치는 요인

표준주의 수행도에 영향을 미치는 요인을 분석하기 위해 총 4개 모델로 구성된 위계적 회귀분석을 실시하였다(Table 5). 분석에 앞서 표준주의 수행도에 유의한 차이가 확인된 일반적 특성 중 결혼 여부, 최종 학력, 임상근무경력, 일평균 근무시간과 주요 변수인 표준주의 인지도, 감염관리 조직문화 및 감염예방환경을 독립변수로 설정하였다. 결혼 여부 중 ‘미혼’, 일평균 근무시간 중 ‘8∼9시간’, 최종 학력 중 ‘전문학사’는 가변수 처리하였으며, 임상근무경력은 표준주의 수행도에 미치는 영향을 보다 면밀히 확인하고자 응답된 실제 근무 개월 수를 연속변수로 분석에 포함하였다.

위계적 회귀분석을 시행하기에 앞서 회귀모형의 적합도를 P-P 도표로 확인하였고 잔차가 45도 기준선에 근접하여 분포하여 정규분포성을 만족하였다. 표준화 잔차 산점도에서 잔차가 모두 0을 중심으로 무작위적으로 분포되어 있어 모형의 선형성과 등분산성 가정을 만족하였다. 이에 본 회귀모형은 적합한 것으로 확인되었다. 또한 Durbin-Watson 값은 1.59로 자기상관이 없는 것으로 판단되는 기준 범위(1.5∼2.5) 내에 포함되어 오차항의 독립성 가정을 충족하였다. 다중공선성 검증 결과 공차한계(tolerance)는 0.31∼0.91로 0.1 이상이었으며 분산팽창요인(variance inflation factor)은 1.02∼3.21으로 10 미만의 범위에 있어 다중공선성의 문제가 없음을 확인하였다. Cook’s distance 값은 1.0 이상이 없어 영향력이 과도한 이상치가 없음이 확인되었다.

모델 1은 일반적 특성에서 유의한 차이가 확인된 결혼 여부, 최종 학력, 임상근무경력, 일평균 근무시간을 투입하였고, 모델 2에서는 표준주의 인지도, 모델 3에서는 감염관리 조직문화, 모델 4에서는 감염예방환경을 추가로 투입하였다. 일반적 특성을 투입한 모델 1에서 회귀모형은 통계적으로 유의한 것으로 나타났으며(F = 4.57, *p* < .001) 표준주의 수행도에 대한 설명력은 10.0%로 나타났다. 분석 결과, 최종 학력이 ‘전문학사’인 집단보다 ‘석사’(β = .26, *p* = .047)인 집단에서, 그리고 일평균 근무시간은 ‘8∼9시간’ 집단보다 ‘8시간 미만’(β = .21, *p* = .005) 집단에서 표준주의 수행도가 높은 것으로 확인되었다. 표준주의 인지도를 추가로 투입한 모델 2는 설명력이 23.0% 유의하게 증가하여 총 33.0%의 설명력을 보였다(F = 14.40, *p* < .001). 모델 2에서는 표준주의 인지도(β = .49, *p* < .001)가 높을수록, 그리고 일평균 근무시간이 ‘8∼9시간’ 집단에 비해 ‘8시간 미만’(β = .15, *p* = .022)인 경우 표준주의 수행도에 유의한 영향을 미치는 것으로 나타났다. 감염관리 조직문화를 추가로 투입한 모델 3은 설명력이 19.0% 증가하였으며, 총 52.0%의 설명력을 나타냈다(F = 25.57, *p* < .001). 분석 결과, 감염관리 조직문화(β = .46, *p* < .001), 표준주의 인지도(β = .38, *p* < .001), 임상근무경력(β = .18, *p* = .008)이 표준주의 수행도에 영향을 미치는 요인으로 확인되었다. 마지막으로 감염예방환경을 추가로 투입한 모델 4는 설명력에 유의한 변화가 나타나지 않았다. 최종 분석 결과, 표준주의 수행도에 영향을 미치는 요인은 감염관리 조직문화(β = .41, *p* < .001), 표준주의 인지도(β = .37, *p* < .001), 임상근무경력(β = .18, *p* = .007) 순으로 나타났으며 최종 모델의 설명력은 52.0%이었다(F = 23.39, *p* < .001).

## 논의

본 연구는 중환자실 간호사의 표준주의 수행에 영향을 미치는 요인을 개인적, 조직적, 환경적 측면에서 통합적으로 분석하고 주요 영향 요인을 규명하고자 시도되었다. 연구 결과, 중환자실 간호사의 표준주의 인지도와 수행도는 모두 높은 수준이었으나 상대적으로 감염관리 조직문화 인식 수준은 다소 낮은 것으로 확인되었다. 이에 다음과 같이 주요 연구 결과에 대해 논의해보고자 한다.

본 연구에서 중환자실 간호사의 표준주의 인지도는 5점 만점에 평균 4.80점으로 높은 수준이었다. 세부 항목 중 ‘안전한 주사행위’가 4.93점으로 가장 높은 점수로 나타났다. 이는 병원간호사회의 근거기반 임상간호실무지침 정맥주입요법, 식품의약품안전처의 주사제 안전사용 가이드라인 등 각종 지침을 기반으로 한 안전주사실무가 임상현장에 안정적으로 정착되었음을 시사한다. 반면, ‘환경관리’ 항목이 4.65점으로 가장 낮은 점수가 나타났다. 이는 앞선 결과에 비추어 볼 때 중환자실 간호사가 환경관리 감독 업무보다 환자에게 제공하는 직접 술기 위주의 간호를 표준주의의 우선적인 항목으로 인지하고 있는 것으로 해석된다. 다만 본 연구에서 사용된 표준주의 인지도 측정 도구는 선행연구의[28] 표준주의 수행도 측정 도구를 바탕으로 본 연구자가 수정·보완한 것으로, 해당 도구를 사용하여 표준주의 인지도를 확인한 선행연구가 부재하여 결과 해석에 제한점이 있다. 그럼에도 불구하고 의료관련감염의 상당 부분이 의료진의 손과 환경에 기인한다는 점은[2,3] 철저한 환경관리의 필요성을 시사한다[2,38]. 이에 의료환경 청결 유지에 대한 책임과 감독의 의무를 지닌 간호사를 대상으로 환경관리 인식을 제고할 수 있는 교육과 캠페인 등 다양한 실천 전략을 마련할 필요가 있다.

본 연구에서 중환자실 간호사의 표준주의 수행도는 5점 만점에 평균 4.56점이었다. 이는 앞서 분석한 표준주의 인지도 평균 점수 4.80점과 비교할 때 상대적으로 낮은 수준이다. 본 연구에서 사용된 도구는 선행연구와 문항 구성과 문항 수에 차이가 있어 직접적인 수치 비교에는 제한이 있으나, 간호사의 표준주의 인지 수준에 비해 실제 수행도가 낮게 나타나는 경향은 기존의 연구 결과들과 맥락을 같이한다[5,6,18,19]. 표준주의 수행도 측정 항목 중 ‘안전한 주사행위(4.88점)’는 가장 점수가 높은 항목으로 이는 표준주의 인지도 항목과 동일하였다. 반면 점수가 가장 낮은 항목은 ‘호흡기 예절(4.29점)’로 여러 선행연구에서 ‘개인보호구’ 항목의 점수가 가장 낮은 점과[7,10] 차이가 있었다. 이러한 차이는 2015년 중동호흡기증후군 유행과 2019년 COVID-19 이후 개인보호구의 중요성이 강조됨에 따라 인식과 수행 수준이 향상되었기 때문으로 해석된다. 반면 ‘호흡기 예절’ 항목의 점수가 가장 낮게 나타난 것은 본 도구의 구성적 특성과 중환자실의 특수한 환경에 기인한 것으로 생각된다. 해당 영역은 간호사 자신의 호흡기 예절뿐 아니라 환자에게 호흡기 예절을 교육하거나 마스크를 제공하는 문항이 포함되어 있다. 그러나 중환자실은 의식 저하나 인공호흡기 적용으로 인해 의사소통 및 교육이 어려운 환자가 많으므로 관련 중재를 수행할 기회가 적어 해당 항목의 점수가 낮게 산출된 것으로 보인다.

감염관리 조직문화 점수는 5점 만점에 평균 4.02점이었으며 7점 척도를 사용한 선행연구와의 비교를 위하여 선형변환공식을 적용하여 점수를 환산하였다. 그 결과 7점 만점에 5.53점이 산출되었으며 이는 COVID-19 이전 선행연구에서 나타난 평균 5.51점과 유사한 수준이었다[13,25]. 비록 척도 환산 결과의 해석에는 한계가 존재하나, 주목할 점은 감염관리 조직문화 중 의사소통 관련 문항인 ‘감염관리지침이 잘 지켜지지 않는다는 것을 알았을 때 부서에 자유롭게 의견을 제시한다(3.52점).’가 가장 낮은 점수를 보였다는 것이다(Appendix 3). 이러한 결과는 COVID-19 이후 의료현장에서 감염관리를 위한 물리적·제도적 지원 체계는 개선되었음에도[2] 이를 뒷받침하는 관리자의 태도나 부서 내 의사소통 체계 등에는 유의미한 변화가 없었음을 시사한다. 이로 인해 감염관리 지침 위반 사항을 발견하더라도 구성원 간에 이를 자유롭게 공유하거나 개선을 요구하기 어려운 조직적 분위기가 존재하는 것으로 풀이된다.

한편, 감염예방환경에 대한 인식 수준은 5점 만점에 평균 4.42점으로 동일한 측정 도구를 이용한 선행연구의 평균 4.04점보다 다소 높은 결과가 나타났다[11,29]. 이는 부적절한 감염예방환경의 원인을 개인보호구의 부족으로 보고한 선행연구와 달리[10,11], 본 연구에서는 개인보호구 항목 중 ‘감염노출 방어를 위한 개인보호구(마스크, 안면보호대, 글러브)가 다양하게 구비되어 있다(4.80점).’ 문항의 점수가 가장 높은 것과(Appendix 4) 관련이 있어 보인다. 이러한 긍정적인 변화는 2015년 중동호흡기증후군 유행과 2020년 COVID-19를 거치면서 국가 차원의 감염병 대응 물리적·제도적 지원 체계가 강화된 결과로 해석해 볼 수 있겠다[2]. 반면, 감염예방환경 측정 항목 중 ‘우리병원은 감염 예방교육을 정기적으로 실시한다(4.05점).’ 문항의 점수가 가장 낮게 나타났다(Appendix 4). 이는 본 연구의 자료 수집 시점인 2023년 12월 당시 온라인 중심의 비대면 교육이 보편화되었던 시기적 상황이 반영된 결과로 생각된다. 온라인 교육은 대면 교육과 비교했을 때 물리적, 심리적 경계가 유연하여 학습자가 직무 교육과 사생활의 영역을 구획하는데 어려움을 초래할 수 있다[39]. 의료기관 내 직무 관련 온라인 교육은 별도의 교육 시간을 보장하지 않고 개인 시간을 할애해야 하는 경우가 빈번하다. 이로 인해 간호사는 온라인 직무 교육을 공식적인 조직 시스템으로 인식하기보다, 사생활을 침범하는 비정형적 부담으로 지각하게 되어[9] 결과적으로 교육의 정기적 운영에 대한 체감도가 저하된 것으로 풀이된다. 그러나 온라인 교육은 시공간적 제약을 극복하여 교육의 연속성을 유지하고 대면 교육에 상응하는 학업성취도를 보이는 강점이 존재한다[40]. 이러한 온라인 교육의 효율성을 유지하면서도 학습 몰입도를 높일 수 있도록 실시간 토론, 소규모 그룹 과제, 실물 수업 자료 제공 등 추가 전략을 병행하여 교육 효과를 보완할 필요가 있다.

본 연구에서 중환자실 간호사의 표준주의 수행도에 영향을 미치는 다각적 측면의 요인을 분석한 결과, 조직적 요인인 감염관리 조직문화가 가장 강력한 영향 요인으로 확인되었으며 개인적 요인인 표준주의 인지도, 그리고 임상근무경력이 그 뒤를 이었다. 반면 환경적 요인인 감염예방환경은 통계적으로 유의하지 않은 것으로 나타났다. 이는 중환자실 간호사의 표준주의 수행도를 향상하기 위해서는 개인적 차원의 노력과 더불어 이를 지지하고 촉진하는 조직 차원의 기반 조성이 핵심적임을 시사한다.

구체적으로 살펴보면, 가장 큰 영향력을 보인 감염관리 조직문화는 구성원이 이를 긍정적으로 인식할수록 표준주의 수행도가 향상된다는 선행연구 결과를[6,13,25] 지지하였다. 특히 주목할 점은 위계적 회귀모형의 단계별 변화에서 감염관리 조직문화가 투입된 모델 3의 설명력이 19.0% 대폭 상승했다는 사실이다. 이는 감염관리 조직문화가 간호사 개인의 신념이나 습관과 같은 내부적인 요소를 실제 표준주의 수행 행동으로 전환시키는 결정적인 역할로 작용함을 의미한다[40,41]. 반면 감염관리 조직문화 점수가 COVID-19 전후로 큰 변화가 없다는 사실은 건설적인 의사소통이 간호사의 표준주의 수행률을 향상하고 의료관련감염 발생 감소에 긍정적 영향을 미친다는 선행연구 결과에[13,24] 비추어 볼 때 비판적으로 검토될 필요가 있다. 즉, 감염관리 조직문화 개선을 실제 표준주의 수행으로 연결하기 위해서는 오류나 지침 미준수 사례를 비난 없이 공유할 수 있는 지지적이며 건설적인 비처벌적 의사소통 체계가[42] 마련되어야 한다. 이와 더불어 간호사 주도의 표준화된 감염관리 프로토콜 제작과 체계적인 접근은 의료관련감염 감소에 효과적이므로[31,43], 표준주의 수행 향상을 위한 조직적 차원의 시스템 구축이 병행되어야 한다. 다만 감염관리와 관련된 조직적 체계가 변화하는 과정에서 구성원은 새로운 지침 이행에 대한 부담을 느끼고 기존의 익숙한 관행을 고수하려는 경향을 보일 수 있다[44]. 따라서 감염관리 조직문화 개선은 단기간의 급진적 전환보다는[44] 부서 내 비처벌적 의사소통 체계 수립, 관리자의 지지적 리더십 구축 등을 토대로[42] 점진적인 변화를 도모하는 것이 바람직해 보인다.

두 번째 영향 요인은 표준주의 인지도였으며 이는 개인의 내적 특성이 행동을 결정하는 일차적인 근거가 된다는 이론적 기틀을[9] 뒷받침하는 결과이다. 눈여겨볼 점은 간호사의 표준주의 인지 수준에 비해 수행도가 낮게 나타나는 경향이[5-7,18,19] 반복된다는 점이다. 이는 표준주의에 대한 ‘인지’가 ‘수행’ 수준으로 직결되지 않으며 인지가 실제 행동으로 전환되는 과정에서 조직적, 환경적 측면 등 다차원적인 제약 요인이 존재함을 시사한다[5,20]. 표준주의 인지도는 표준주의 수행을 위한 필수 전제 조건이나 복잡한 임상 현장에서 높은 수준의 수행도를 일관되게 유지하는 데에는 한계가 있다. 따라서 간호사의 인지 수준을 행동으로 이끌어내기 위한 심층적인 고찰과 다각적 개입이 필요할 것으로 판단된다.

세 번째 영향 요인으로 임상근무경력이 확인되었으며 이러한 결과는 선행연구와[6,14-16] 일맥상통하였다. 이는 간호사의 임상 경력이 증가할수록 풍부한 경험과 축적된 전문성을 기반으로 업무의 숙련도가 높아져[6] 표준주의 원칙을 핵심 우선순위로 내면화한 결과로 풀이된다. 이처럼 내면화된 표준주의 원칙이 실제 임상 현장에서 이행될 수 있도록 워크숍, 세미나, 컨퍼런스 등을 통해 개인의 전문성을 구체화하는 교육적 중재가 요구된다[45]. 다만 본 연구결과에서 확인된 바와 같이 개인적 요인만으로 표준주의 수행을 향상하기에는 한계가 있다. 이에 지식 전달 교육과 함께 개인의 내적 역량을 표출할 수 있는 시뮬레이션 기반 실습 교육과 현장 피드백을 강화하고 이를 뒷받침하는 조직적 지원이 유기적으로 병행되어야 한다.

한편 감염예방환경은 표준주의 수행도에 유의한 영향을 미치지 않는 것으로 나타났다. 이는 COVID-19 이후 중환자실의 음압격리병실 시설 기준 강화, 의료종사자 대상 교육 내실화, 장비와 시설의 확충 등 다각적인 노력이 집중되어[46] 실질적인 물리적·제도적 지원 체계가 임상 현장에 안정적으로 정착된 결과로 해석된다. 이러한 결과는 감염예방환경이 표준주의 수행을 위한 기초적인 자산으로서, 간호사의 표준주의 수행도 차이를 결정짓는 변별 요인으로의 기능이 약화됨을 시사한다. 다만 감염예방교육의 정기적 운영에 대한 낮은 교육 체감도는 향후 질적 개선을 위한 추가적인 전략이 필요함을 보여준다. 동시에 본 연구는 설비와 시스템이 유사한 동일 종별의 의료기관 두 곳만을 대상으로 하였기에 환경적 요인의 변동 폭이 작아 통계적 유의성이 나타나지 않았을 가능성을 배제할 수 없다. 감염예방환경은 단발적인 구축보다는 지속적인 유지와 관리가 중요하므로[41] 의료환경의 안전한 감염예방환경 유지를 위해 의료종사자 개인의 실천 노력뿐 아니라 의료기관의 제도적 지원, 나아가 국가 차원의 정책적 뒷받침이 지속되어야 한다.

본 연구는 연구대상자 모집 시 연령에 제한을 두지 않았음에도 연구대상자의 연령대가 20∼30대에 편중되어 있으며 이러한 연령대 분포는 상급종합병원의 인력 구조적 특성이 반영된 것으로[47] 판단된다. 더불어 연구대상자를 편의표집 방식으로 선정하였으므로 연구결과를 전체 간호사 집단으로 일반화하기에 제한점이 있다. 또한 자가 보고식 설문 조사를 통해 자료를 수집하였기에 연구대상자의 사회적 바람직성 편향이 개입되어 실제 표준주의 수행 수준보다 연구 결과가 긍정적으로 보고되었을 가능성이 있다.

이러한 제한점에도 불구하고 본 연구는 다음과 같은 의의를 가진다. 첫째, 본 연구는 간호사의 표준주의 수행도에 영향을 미치는 요인을 개인적, 조직적, 환경적 측면에서 다각적으로 분석하였다. 둘째, COVID-19 이후 변화된 의료환경에서 간호사의 표준주의 수행 현황을 파악하여 향후 관련 연구와 교육 중재를 위한 기초자료를 제공하였다. 셋째, 병원체의 생물학적 전파를 차단하여 환자 안전을 확보한다는 최우선 목표를 달성하기 위해 물리적 설비 중심의 접근을 넘어 인적·조직적 역량 강화로 감염관리의 방향을 확장해야 할 기초간호학적 근거를 제시하였다.

## 결론

본 연구는 상급종합병원 중환자실에 근무하는 간호사를 대상으로 COVID-19 이후 변화된 임상 현장에서 표준주의 수행도에 영향을 미치는 요인을 개인적, 조직적, 환경적 측면에서 통합적으로 규명하고자 수행되었다. 위계적 회귀분석을 시행한 결과, 표준주의 수행도를 설명하는 최종 모델의 전체 설명력은 52.0%로 나타났다. 표준주의 수행도에 유의한 영향을 미치는 요인은 조직적 측면의 감염관리 조직문화, 개인적 측면의 표준주의 인지도와 임상근무경력 순으로 확인되었다. 환경적 측면의 감염예방환경은 통계적으로 유의한 영향을 미치지 않는 것으로 나타났다. 이는 강화된 물리적·제도적 지원 체계가 임상현장에 안정적으로 정착되었음을 시사하나, 조사 결과 상대적으로 낮게 나타난 감염예방교육의 정기적 운영 체감도는 향후 교육의 접근성과 지속성을 강화할 필요가 있음을 보여준다. 이러한 맥락에서 간호사의 표준주의 수행 향상을 위해서는 지식 전달 교육과 더불어 경력별 맞춤형 중재와 시뮬레이션 기반 실습, 현장 피드백 체계 구축을 통하여 ‘인지’ 수준을 ‘수행’ 수준으로 전환하는 노력이 요구된다. 또한, 감염관리 오류나 지침 미준수 사례를 비난 없이 공유하고 이를 개선의 기회로 삼을 수 있도록 하는 지지적이고 건설적인 비처벌적 의사소통 체계 구축과 관리자의 지지적 리더십 확립 방안 마련이 요구된다.

본 연구 결과를 바탕으로 다음과 같이 제언하고자 한다.

첫째, 본 연구에서 표준주의 수행의 가장 유의한 영향 요인으로 확인된 감염관리 조직문화를 개선하기 위해 비처벌적인 의사소통 체계를 구축하고 간호사 주도의 표준화된 감염관리 프로토콜을 제작하여 임상 현장에 적용하는 후속 연구를 제언한다.

둘째, 본 연구의 제한점을 보완하고 표준주의 수행의 실질적인 저해 요인을 규명하기 위해 연구 방법의 다변화가 필요하다. 다양한 종별의 의료기관과 부서를 대상으로 반복 연구를 실시하여 취약한 수행 항목을 구체화하며, 동시에 인지와 수행의 간극 속에 존재하는 복합적인 맥락을 심층적으로 파악하기 위한 질적 연구를 병행할 것을 제언한다.

셋째, 간호사의 표준주의 인지 수준과 수행도 간의 간극을 해소하기 위해서는 다각적인 개입이 요구된다. 이에 지식 전달 위주의 교육뿐 아니라 간호사의 임상 경력별 특성을 반영하고 개인의 내적 역량을 표출할 수 있도록 하는 시뮬레이션 기반의 실습 교육과 현장 피드백 체계를 구축하여 그 효과를 검증하는 중재 연구를 제언한다.

## Figures and Tables

**Table 1.** General Characteristics of Participants (N = 166)

Characteristics	Categories	M ± SD	n (%)
Age (years)	< 30		80 (48.2)
	≥ 30		86 (51.8)
Sex	Men		14 (8.4)
	Women		152 (91.6)
Marital status	Married		48 (28.9)
	Single		118 (71.1)
Education level	Associate’s degree		10 (6.0)
	Bachelor’s degree		135 (81.3)
	Master’s degree		21 (12.7)
Clinical experience (months)		73.49 ± 37.25	
	6–23		19 (11.4)
	24–59		51 (30.7)
	60–119		66 (39.8)
	≥ 120		30 (18.1)
Type of ICU	Surgical		85 (51.2)
	Medical		61 (36.7)
	Emergency		20 (12.1)
Number of patients per day	< 3		157 (94.6)
	3–4		9 (5.4)
Average daily working hours	< 8		15 (9.0)
	8–9		151 (91.0)
Exposure to infectious agents	Yes		75 (45.2)
	No		91 (54.8)

M = Mean; SD = Standard deviation; ICU = Intensive care unit.

**Table 2.** Levels of Awareness and Performance of Standard Precautions, Organizational Culture for Infection Control, and Infection Prevention Environment (N = 166)

Variables	M ± SD	Min	Max
Awareness of SPs	4.80 ± 0.25	3.92	5.00
Hand hygiene	4.76 ± 0.30	3.90	5.00
Personal protective equipment	4.81 ± 0.28	3.89	5.00
Respiratory etiquette	4.77 ± 0.40	3.33	5.00
Patient care equipment	4.88 ± 0.28	4.00	5.00
Care of the environment	4.65 ± 0.49	3.00	5.00
Linen	4.67 ± 0.48	3.00	5.00
Safe injection practices	4.93 ± 0.22	3.40	5.00
Worker safety	4.86 ± 0.29	3.75	5.00
Patient assignment	4.85 ± 0.33	4.00	5.00
Performance of SPs	4.56 ± 0.39	3.03	5.00
Hand hygiene	4.48 ± 0.51	2.60	5.00
Personal protective equipment	4.54 ± 0.43	2.89	5.00
Respiratory etiquette	4.29 ± 0.68	2.00	5.00
Patient care equipment	4.77 ± 0.44	3.00	5.00
Care of the environment	4.37 ± 0.81	1.00	5.00
Linen	4.46 ± 0.66	2.50	5.00
Safe injection practices	4.88 ± 0.31	2.80	5.00
Worker safety	4.62 ± 0.47	3.00	5.00
Patient assignment	4.56 ± 0.63	2.50	5.00
Organizational culture for infection control	4.02 ± 0.53	2.80	5.00
Infection prevention environment	4.42 ± 0.47	3.27	5.00

M = Mean; SD = Standard deviation; Min = Minimum; Max = Maximum; SPs = Standard precautions.

**Table 3.** Differences in Performance of Standard Precautions by Participants’ General Characteristics (N = 166)

Variables	Categories	Performance of SPs	
		M ± SD	t or F(*p*) Scheffé
Age (years)	< 30	4.51 ± 0.45	-1.52 (.130)
	≥ 30	4.60 ± 0.32	
Sex	Men	4.49 ± 0.38	-0.72 (.476)
	Women	4.56 ± 0.39	
Marital status	Married	4.66 ± 0.33	2.26 (.025)
	Single	4.51 ± 0.41	
Education level	Associate’s degree^a^	4.30 ± 0.58	4.86 (.009)
	Bachelor’s degree^b^	4.55 ± 0.38	a,b < c
	Master’s degree^c^	4.75 ± 0.26	
Clinical experience (months)	6–23^a^	4.48 ± 0.42	3.30 (.022)
	24–59^b^	4.54 ± 0.46	a,b,c < d
	60–119^c^	4.50 ± 0.35	
	≥ 120^d^	4.75 ± 0.24	
Type of ICU	Surgical	4.51 ± 0.43	2.07 (.130)
	Medical	4.57 ± 0.37	
	Emergency	4.70 ± 0.18	
Number of patients per day	< 3	4.57 ± 0.37	1.42 (.191)
	3–4	4.30 ± 0.57	
Average daily working hours	< 8	4.83 ± 0.17	5.54 (< .001)
	8–9	4.53 ± 0.40	
Exposure to infectious agents	Yes	4.52 ± 0.38	-1.00 (.318)
	No	4.58 ± 0.40	

M = Mean; SD = Standard deviation; SPs = Standard precautions; ICU = Intensive care unit.

**Table 4.** Correlations among Variables (N = 166)

Variables	Awareness of SPs	Infection prevention environment	Organizational culture for infection control
	r (*p*)		
			
Infection prevention environment	.37 (< .001)	1	-
Organizational culture for infection control	.27 (< .001)	.70 (< .001)	1
Performance of SPs	.55 (< .001)	.52 (< .001)	.58 (< .001)

SPs = Standard precautions.

**Table 5.** Factors Influencing the Performance of Standard Precautions (N = 166)

Variables	Model 1				Model 2				Model 3				Model 4			
	B	SE	β	*p*	B	SE	β	*p*	B	SE	β	*p*	B	SE	β	*p*
(Constant)	4.52	0.16		< .001	0.88	0.51		.041	.26	0.44		.394	0.34	0.46		.433
Marital Status (married) (ref. single)	0.06	0.07	.07	.431	0.04	0.06	.05	.499	0.03	0.05	.04	.547	0.03	0.05	.04	.551
Education level (bachelor’s degree) (ref. associate’s degree)	0.24	0.12	.24	.052	0.12	0.11	.12	.254	0.07	0.09	.07	.421	0.07	0.09	.07	.430
Education level (master’s degree) (ref. associate’s degree)	0.31	0.15	.26	.047	0.14	0.13	.12	.303	0.06	0.11	.05	.618	0.06	0.11	.05	.615
Clinical experience^†^	0.00	0.00	.15	.107	0.00	0.00	.13	.105	0.00	0.00	.18	.008	0.00	0.00	.18	.007
Average working hours (< 8) (ref. 8–9)	0.29	0.10	.21	.005	0.21	0.09	.15	.022	0.11	0.08	.08	.160	0.18	0.08	.08	.166
Awareness of SPs					0.77	0.10	.49	< .001	0.59	0.09	.38	< .001	0.57	0.09	.37	< .001
Organizational culture for infection control									0.34	0.04	.46	< .001	0.22	0.04	.41	< .001
Infection prevention environment													0.07	0.07	.08	.304
R²	.13				.35				.54				.54			
Adj.R²	.10				.33				.52				.52			
△R²					.23				.19				.00			
F(*p*)	4.57 (< .001)				14.40 (< .001)				25.57 (< .001)				23.39 (< .001)			

SE = Standard error; ref. = Reference; SPs = Standard precautions.

† Clinical experience was treated as a continuous variable (unit: months).

## Appendix 1. Item-level Responses for Awareness of Standard Precautions (N = 166)

**Table:**

Variables	Categories		M ± SD	Min	Max
Hand hygiene	1	Wash hands with soap and water when visible contaminants like blood or body fluids are present.	4.93 ± 0.27	3	5
	2	Wash hands with soap and water or use hand sanitizer when no visible contaminants are present.	4.83 ± 0.41	3	5
	3	Perform hand hygiene before direct contact with patients.	4.81 ± 0.42	3	5
	4	Perform hand hygiene after contact with blood, body fluids, excretions, mucous membranes, non-intact skin, or wound dressings.	4.95 ± 0.23	4	5
	5	Perform hand hygiene after contact with a patient's intact skin (e.g., measuring pulse/blood pressure, assisting a patient).	4.62 ± 0.57	3	5
	6	Perform hand hygiene when moving from a contaminated body site to a clean body site on the same patient.	4.78 ± 0.43	3	5
	7	Perform hand hygiene after contact with objects or equipment in the patient's vicinity (e.g., tidying the bed, touching a monitor).	4.55 ± 0.60	3	5
	8	Perform hand hygiene before putting on gloves.	4.52 ± 0.63	3	5
	9	Perform hand hygiene after removing gloves.	4.80 ± 0.43	3	5
	10	Perform hand hygiene after removing a gown.	4.77 ± 0.47	3	5
Personal protective equipment	11	Wear gloves before contact with a patient's blood, body fluids, mucous membranes, or non-intact skin.	4.86 ± 0.40	3	5
	12	Change gloves after contact with a contaminated site before touching a clean site on the same patient.	4.78 ± 0.46	3	5
	13	Change gloves when moving from contact with one patient to another.	4.93 ± 0.26	4	5
	14	Wear a gown when there is a risk of splashing blood, body fluids, secretions, or excretions.	4.89 ± 0.32	4	5
	15	Remove the gown before leaving the patient's room.	4.78 ± 0.43	3	5
	16	When removing a gown, consider the front contaminated and fold it so the inside comes out.	4.70 ± 0.53	3	5
	17	Do not reuse a gown even for the same patient.	4.76 ± 0.48	3	5
	18	Use a mask when there is a possibility of splashing blood, body fluids, or secretions.	4.91 ± 0.29	4	5
	19	Wear goggles when there is a possibility of splashing blood, body fluids, or secretions.	4.67 ± 0.55	3	5
Respiratory etiquette	20	Educate patients with respiratory symptoms to cover their mouth and nose with tissue when coughing, discard the used tissue, and perform hand hygiene.	4.73 ± 0.54	3	5
	21	Provide masks to patients with a persistent cough.	4.68 ± 0.58	3	5
	22	Always wear a mask while caring for patients if you have respiratory symptoms.	4.90 ± 0.32	3	5
Patient care equipment	23	Discard used needles or sharp instruments in designated sharps containers.	4.95 ± 0.23	4	5
	24	Wear personal PPE when handling contaminated instruments and equipment.	4.81 ± 0.44	3	5
Care of the environment	25	Verify that environmental management staff clean the patient's surroundings and disinfect if heavily contaminated.	4.56 ± 0.59	3	5
	26	Verify that environmental management staff wipe the bed, table, etc., with disinfectant after the patient is discharged.	4.73 ± 0.48	3	5
Linen	27	Separate and process linen contaminated with blood, body fluids, excretions, or secretions from general laundry.	4.66 ± 0.53	3	5
	28	Place used linen in the laundry hamper carefully to avoid contaminating the surroundings.	4.67 ± 0.52	3	5
Safe injection practices	29	Do not reuse used syringes by only changing the needle.	4.93 ± 0.25	4	5
	30	Consider IV fluid sets used on patients as contaminated and discard them.	4.94 ± 0.26	3	5
	31	Do not mix medications for multiple patients with a single syringe when mixing diluents.	4.94 ± 0.26	3	5
	32	Do not draw multiple doses from single-dose vials or ampules; discard remaining medication immediately.	4.90 ± 0.32	3	5
	33	Disinfect the rubber stopper of multi-dose vials every time before use, and use sterile syringes/needles.	4.93 ± 0.27	3	5
Worker safety	34	Be careful to avoid needle-stick or sharps injuries when handling sharp instruments.	4.95 ± 0.21	4	5
	35	Discard used needles without recapping.	4.83 ± 0.43	3	5
	36	Do not bend or break needles.	4.80 ± 0.49	2	5
	37	When performing CPR, avoid mouth-to-mouth and wear gloves and a mask.	4.89 ± 0.34	3	5
Patient assignment	38	If an infected patient is in a multi-patient room, assign them to a room with patients having the same disease.	4.81 ± 0.41	3	5
	39	Place the patient in a single room if there is a high risk of transmission or if symptoms are progressing.	4.87 ± 0.35	3	5

M = Mean; SD = Standard deviation; Min = Minimum; Max = Maximum; PPE = Personal protective equipment; IV = Intravenous; CPR = Cardiopulmonary resuscitation.

## Appendix 2. Item-level Responses for Performance of Standard Precautions (N = 166)

**Table:**

Variables	Categories		M ± SD	Min	Max
Hand hygiene	1	Wash hands with soap and water when visible contaminants like blood or body fluids are present.	4.84 ± 0.43	2	5
	2	Wash hands with soap and water or use hand sanitizer when no visible contaminants are present.	4.56 ± 0.64	2	5
	3	Perform hand hygiene before direct contact with patients.	4.23 ± 0.83	2	5
	4	Perform hand hygiene after contact with blood, body fluids, excretions, mucous membranes, non-intact skin, or wound dressings.	4.89 ± 0.39	2	5
	5	Perform hand hygiene after contact with a patient's intact skin (e.g., measuring pulse/blood pressure, assisting a patient).	4.42 ± 0.70	2	5
	6	Perform hand hygiene when moving from a contaminated body site to a clean body site on the same patient.	4.30 ± 0.92	1	5
	7	Perform hand hygiene after contact with objects or equipment in the patient's vicinity (e.g., tidying the bed, touching a monitor).	4.22 ± 0.86	2	5
	8	Perform hand hygiene before putting on gloves.	3.98 ± 1.04	1	5
	9	Perform hand hygiene after removing gloves.	4.67 ± 0.62	1	5
	10	Perform hand hygiene after removing a gown.	4.66 ± 0.61	2	5
Personal protective equipment	11	Wear gloves before contact with a patient's blood, body fluids, mucous membranes, or non-intact skin.	4.73 ± 0.53	2	5
	12	Change gloves after contact with a contaminated site before touching a clean site on the same patient.	4.42 ± 0.84	1	5
	13	Change gloves when moving from contact with one patient to another.	4.86 ± 0.44	2	5
	14	Wear a gown when there is a risk of splashing blood, body fluids, secretions, or excretions.	4.72 ± 0.57	2	5
	15	Remove the gown before leaving the patient's room.	4.61 ± 0.63	2	5
	16	When removing a gown, consider the front contaminated and fold it so the inside comes out.	4.65 ± 0.66	2	5
	17	Do not reuse a gown even for the same patient.	4.67 ± 0.65	2	5
	18	Use a mask when there is a possibility of splashing blood, body fluids, or secretions.	4.84 ± 0.44	2	5
	19	Wear goggles when there is a possibility of splashing blood, body fluids, or secretions.	3.40 ± 1.27	1	5
Respiratory etiquette	20	Educate patients with respiratory symptoms to cover their mouth and nose with tissue when coughing, discard the used tissue, and perform hand hygiene.	3.92 ± 1.09	1	5
	21	Provide masks to patients with a persistent cough.	4.05 ± 1.01	1	5
	22	Always wear a mask while caring for patients if you have respiratory symptoms.	4.90 ± 0.39	2	5
Patient care equipment	23	Discard used needles or sharp instruments in designated sharps containers.	4.95 ± 0.28	3	5
	24	Wear PPE when handling contaminated instruments and equipment.	4.60 ± 0.75	2	5
Care of the environment	25	Verify that environmental management staff clean the patient's surroundings and disinfect if heavily contaminated.	4.23 ± 0.87	1	5
	26	Verify that environmental management staff wipe the bed, table, etc., with disinfectant after the patient is discharged.	4.52 ± 0.86	1	5
Linen	27	Separate and process linen contaminated with blood, body fluids, excretions, or secretions from general laundry.	4.30 ± 0.93	1	5
	28	Place used linen in the laundry hamper carefully to avoid contaminating the surroundings.	4.61 ± 0.69	1	5
Safe injection practices	29	Do not reuse used syringes by only changing the needle.	4.92 ± 0.31	3	5
	30	Consider IV fluid sets used on patients as contaminated and discard them.	4.94 ± 0.34	2	5
	31	Do not mix medications for multiple patients with a single syringe when mixing diluents.	4.93 ± 0.27	3	5
	32	Do not draw multiple doses from single-dose vials or ampules; discard remaining medication immediately.	4.79 ± 0.55	2	5
	33	Disinfect the rubber stopper of multi-dose vials every time before use, and use sterile syringes/needles.	4.81 ± 0.53	2	5
Worker safety	34	Be careful to avoid needle-stick or sharps injuries when handling sharp instruments.	4.84 ± 0.42	3	5
	35	Discard used needles without recapping.	4.19 ± 0.91	2	5
	36	Do not bend or break needles.	4.77 ± 0.54	1	5
	37	When performing CPR, avoid mouth-to-mouth and wear gloves and a mask.	4.69 ± 0.66	2	5
Patient assignment	38	If an infected patient is in a multi-patient room, assign them to a room with patients having the same disease.	4.44 ± 0.84	1	5
	39	Place the patient in a single room if there is a high risk of transmission or if symptoms are progressing.	4.65 ± 0.62	2	5

M = Mean; SD = Standard deviation; Min = Minimum; Max = Maximum; PPE = Personal protective equipment; IV = Intravenous; CPR = Cardiopulmonary resuscitation.

## Appendix 3. Item-level Responses for Organizational Culture for Infection Control (N = 166)

**Table:**

	Categories	M ± SD	Min	Max
1	Staff members in the department help each other ensure I can comply with infection control guidelines.	4.33 ± 0.61	3	5
2	Complying with the hospital's infection control guidelines is a natural duty of the department.	4.70 ± 0.51	3	5
3	The head nurse provides ample praise when nurses comply well with infection control guidelines.	3.63 ± 1.03	1	5
4	My supervisor wants me to handle tasks quickly even if it means skipping guidelines when the workload is heavy. (R)	3.98 ± 1.07	1	5
5	I feel free to provide opinions when I notice that infection control guidelines are not being followed.	3.52 ± 0.85	1	5
6	The department head takes strong action when infection control guidelines are repeatedly violated.	3.84 ± 0.84	2	5
7	Staff members make active efforts to prevent healthcare-associated infections.	4.28 ± 0.67	2	5
8	Effects are measured when changes are attempted to reduce healthcare-associated infections.	3.86 ± 0.91	1	5
9	Evaluations of the department's compliance with infection control guidelines are conducted periodically.	4.32 ± 0.68	3	5
10	Feedback on evaluation results regarding compliance with infection control guidelines is always provided.	3.78 ± 091	1	5

M = Mean; SD = Standard deviation; Min = Minimum; Max = Maximum; R = Reverse-scored item.

## Appendix 4. Item-level Responses for Infection Prevention Environment (N = 166)

**Table:**

	Categories	M ± SD	Min	Max
1	Various PPE (e.g., masks, face shields, gloves) are provided to defend against infection exposure.	4.80 ± 0.50	2	5
2	Hospital administrators show interest in and manage staff exposure to infection.	4.36 ± 0.71	2	5
3	It is difficult to perform proper disinfection because cleaning tools or supplies are insufficient. (R)	4.33 ± 0.85	1	5
4	PPE is available but is located far from the nursing station. (R)	4.24 ± 0.92	1	5
5	Our hospital conducts regular infection prevention education.	4.05 ± 0.71	2	5
6	Our hospital provides instruction guidelines for infection prevention.	4.34 ± 0.70	2	5
7	Our hospital organizes and maintains protocols for infection exposure.	4.33 ± 0.77	2	5
8	Sharp objects are separated and collected in designated containers.	4.75 ± 0.46	3	5
9	Our hospital requires regular health check-ups for infection prevention.	4.43 ± 0.87	1	5
10	Our hospital provides regular vaccinations for infection control.	4.55 ± 0.72	1	5
11	Staff members at our hospital use PPE appropriately.	4.45 ± 0.66	1	5

M = Mean; SD = Standard deviation; Min = Minimum; Max = Maximum; PPE = Personal protective equipment; R = Reverse-scored item.
